# Clinician time used for decision making: a best case workflow study using cardiovascular risk assessments and Ask Mayo Expert algorithmic care process models

**DOI:** 10.1186/s12911-016-0334-z

**Published:** 2016-07-20

**Authors:** Frederick North, Samuel Fox, Rajeev Chaudhry

**Affiliations:** Division of Primary Care Internal Medicine, Mayo Clinic, Rochester, MN USA; Office of Information and Knowledge Management, Mayo Clinic, Rochester, MN USA

**Keywords:** Clinical decision support, Clinical burden, Risk factor assessment, Risk calculation, Care process, Atrial fibrillation, Heart failure, Lipid management, Algorithm, Ask Mayo Expert

## Abstract

**Background:**

Risk calculation is increasingly used in lipid management, congestive heart failure, and atrial fibrillation. The risk scores are then used for decisions about statin use, anticoagulation, and implantable defibrillator use. Calculating risks for patients and making decisions based on these risks is often done at the point of care and is an additional time burden for clinicians that can be decreased by automating the tasks and using clinical decision-making support.

**Methods:**

Using Morae Recorder software, we timed 30 healthcare providers tasked with calculating the overall risk of cardiovascular events, sudden death in heart failure, and thrombotic event risk in atrial fibrillation. Risk calculators used were the American College of Cardiology Atherosclerotic Cardiovascular Disease risk calculator (AHA-ASCVD risk), Seattle Heart Failure Model (SHFM risk), and CHA_2_DS_2_VASc. We also timed the 30 providers using Ask Mayo Expert care process models for lipid management, heart failure management, and atrial fibrillation management based on the calculated risk scores. We used the Mayo Clinic primary care panel to estimate time for calculating an entire panel risk.

**Results:**

Mean provider times to complete the CHA_2_DS_2_VASc, AHA-ASCVD risk, and SHFM were 36, 45, and 171 s respectively. For decision making about atrial fibrillation, lipids, and heart failure, the mean times (including risk calculations) were 85, 110, and 347 s respectively.

**Conclusion:**

Even under best case circumstances, providers take a significant amount of time to complete risk assessments. For a complete panel of patients this can lead to hours of time required to make decisions about prescribing statins, use of anticoagulation, and medications for heart failure. Informatics solutions are needed to capture data in the medical record and serve up automatically calculated risk assessments to physicians and other providers at the point of care.

## Background

Clinicians are under an increasing time burden to adequately care for patients. A study from over a decade ago showed that it would take over seven hours per working day for primary care physicians to satisfy preventive service recommendations for their patients [1]. More recently, the most commonly reported barrier to coronary heart disease (CHD) risk assessment was that it was too time consuming [2].

We know that when clinicians are given patients’ risks without having to calculate it themselves, there can be a significant change in management. For example, providing clinicians with a precalculated Framingham risk score resulted in increasing statin prescriptions by 32 % for those with high risk [3]. In the same study, fewer than 20 % of primary care providers routinely calculated 10 year cardiovascular risks in their patients [3].

Currently, a number of guidelines depend on risk calculations. For example, the guideline for primary stroke prevention in those with atrial fibrillation requires calculation of the CHA_2_DS_2_VASc score [4]. The CHA_2_DS_2_VASc score is used to determine future stroke risk to balance against the risk of anticoagulant therapy. Because anticoagulant therapy is associated with hemorrhagic risk, the stroke risk calculation is needed to decide whether the risk of stroke outweighs the risk of use of an antithrombotic. Likewise, the American College of Cardiology guideline to reduce atherosclerotic cardiovascular disease requires evaluating the 10 year atherosclerotic cardiovascular risk [5]. Other risk calculations are also incorporated into guidelines including the FRAX calculator for osteoporosis treatment [6, 7].

Recommendations for complex disease treatment are also incorporating risk calculators. The Seattle Heart Failure Model can be used to assess risk for sudden death in heart failure [8, 9]. Patients making the decision for an implantable cardioverter-defibrillator (ICD) need to know specifics risks, and the risk for death can be powerful information for shared decision making [10–12].

The objective of this study was to examine clinician time involved in risk calculation and decision making. This was done in a setting to estimate the minimum time it might take a provider at the point of care.

## Methods

### Setting

This study took place at Mayo Clinic, Rochester, Minnesota where the electronic health record (EHR) has been in place for over 20 years. Currently, the EHR contains essentially all the data elements required to generate a CHA_2_DS_2_VASc score, the ASCVD risk, and sudden death risk based on the Seattle Heart Failure Model. The EHR includes patient generated data such as tobacco use, as well as laboratory information such as cholesterol levels.

**Fig. 1:**
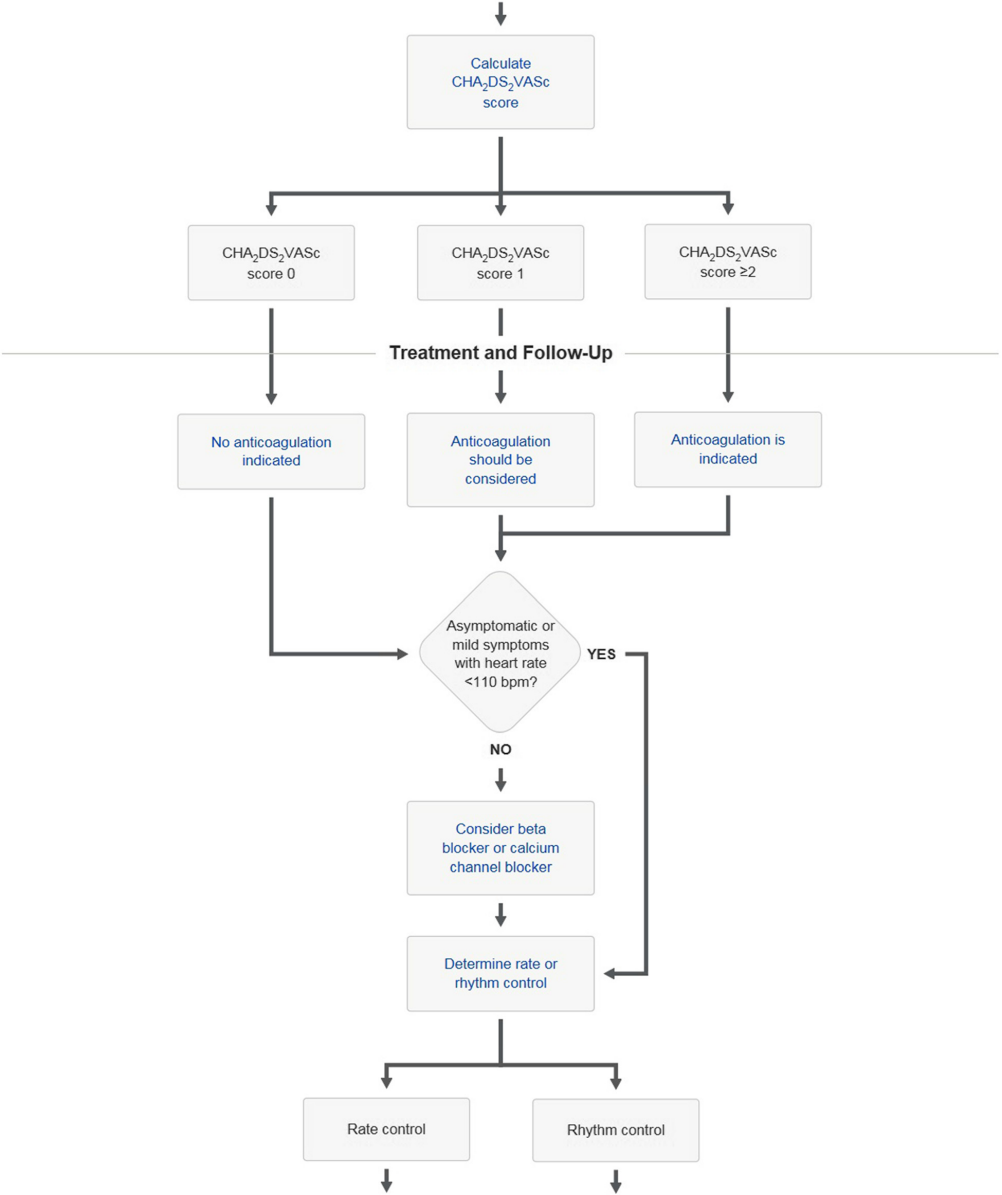
Section of atrial fibrillation care process model from Ask Mayo Expert

**Fig. 2:**
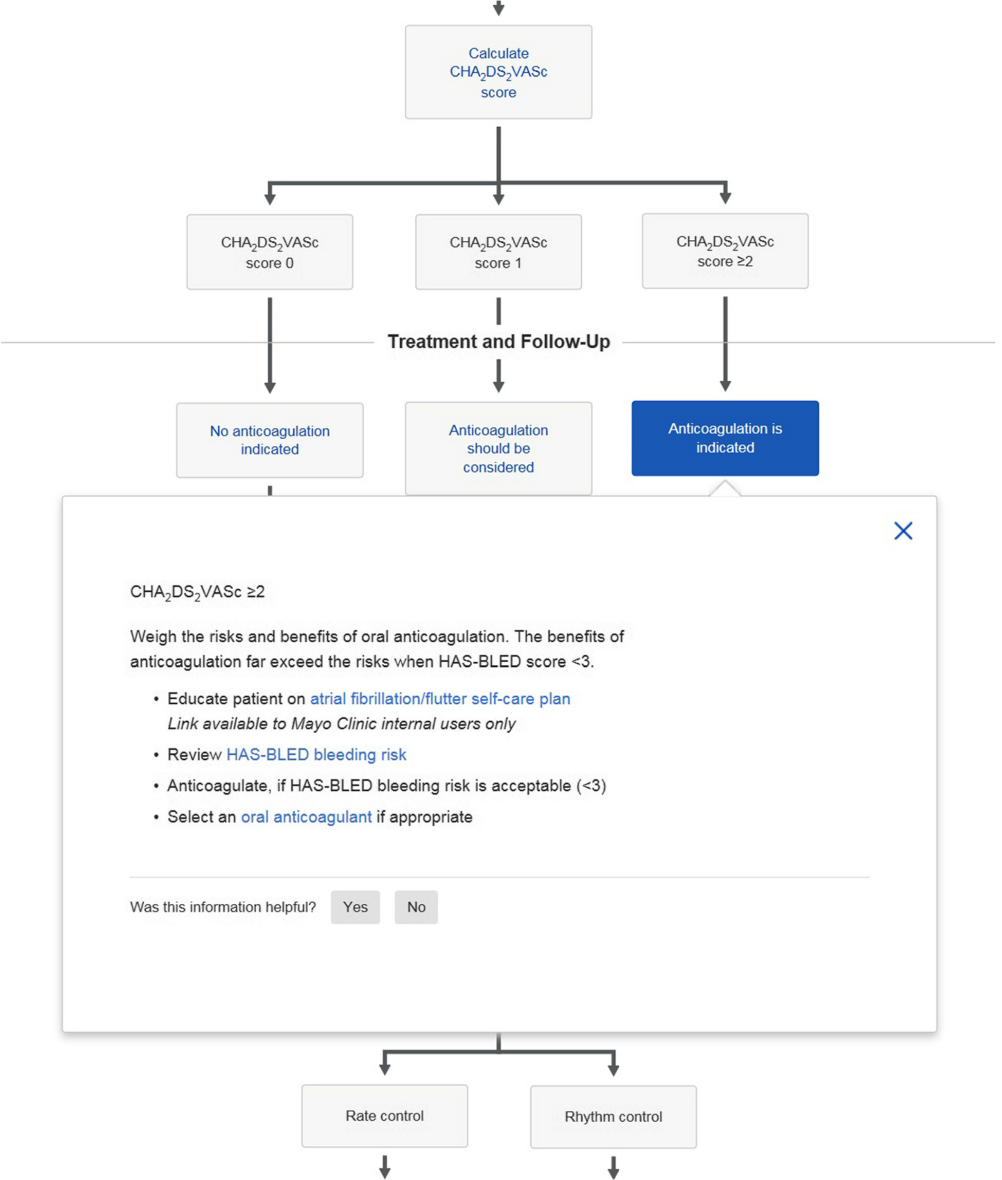
Section of atrial fibrillation care process model from Ask Mayo Expert. Intermediate care point has been expanded showing link to HAS-BLED calculator

Mayo Clinic has a proprietary medical knowledge system called Ask Mayo Expert (Mayo Clinic, Rochester, MN, USA) that is used at the point of care by both specialists and primary care providers. Ask Mayo Expert is a concise, online resource that contains Mayo Clinic-vetted medical knowledge on nearly 2,000 medical topics. In addition, Ask Mayo Expert contains several hundred care process models that contain algorithmic flow diagrams used to support clinical decision making. The flow diagrams in the care models indicate how management of a particular disease or symptom should proceed. The care process models in Ask Mayo Expert range from atrial fibrillation (Figs. 1 and 2) to warts. With flow diagrams in digital format, the decision points, intermediate points, and end points of the care flow can be expanded to show more detailed information (Figs. 1 and 2). Ask Mayo Expert is available to any Mayo provider via the Mayo Intranet and is available to other providers within the Mayo Clinic Care Network.

### Selection of care process models

**Fig. 3:**
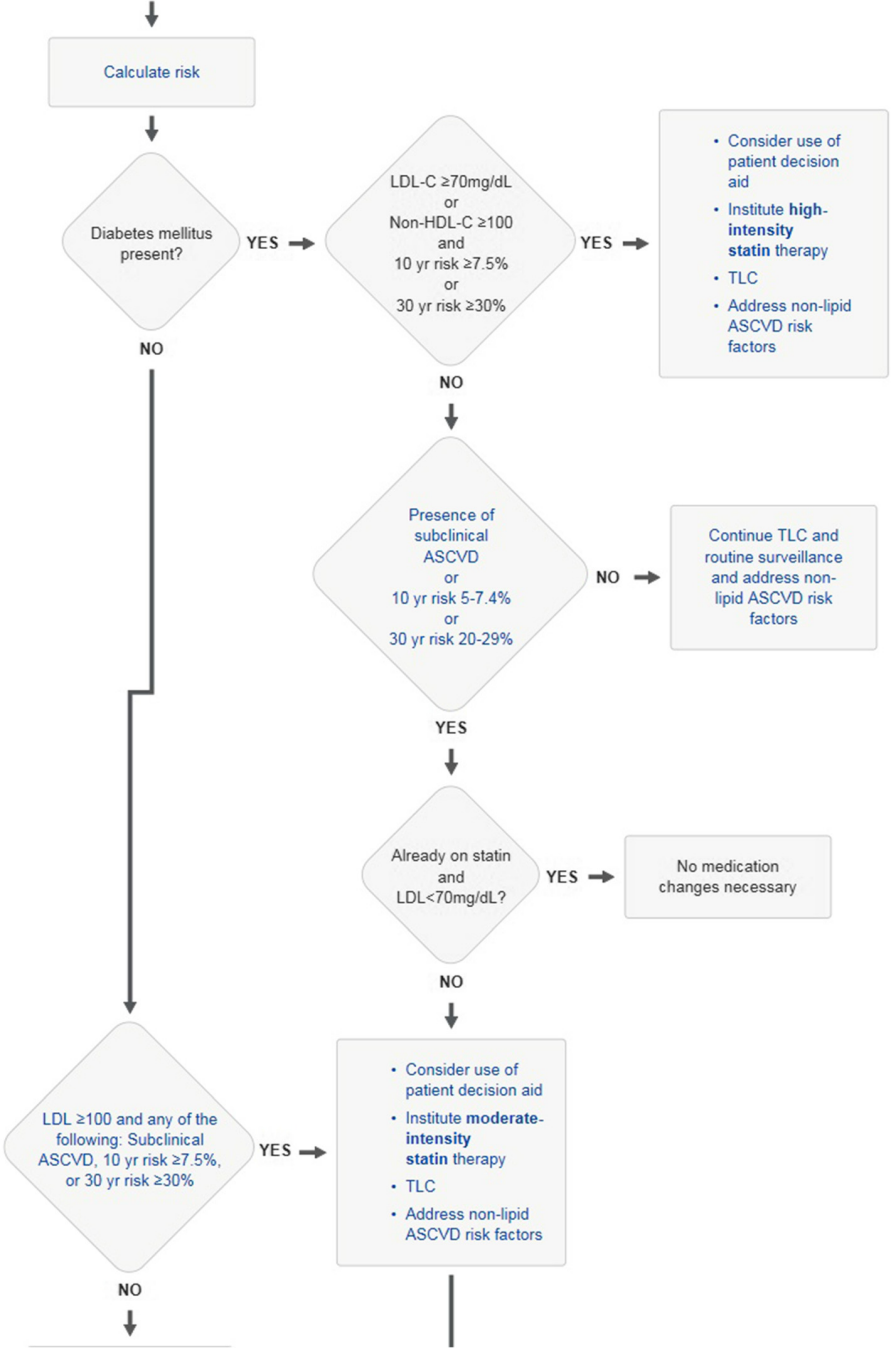
Section of lipid management care process model from Ask Mayo Expert

**Fig. 4:**
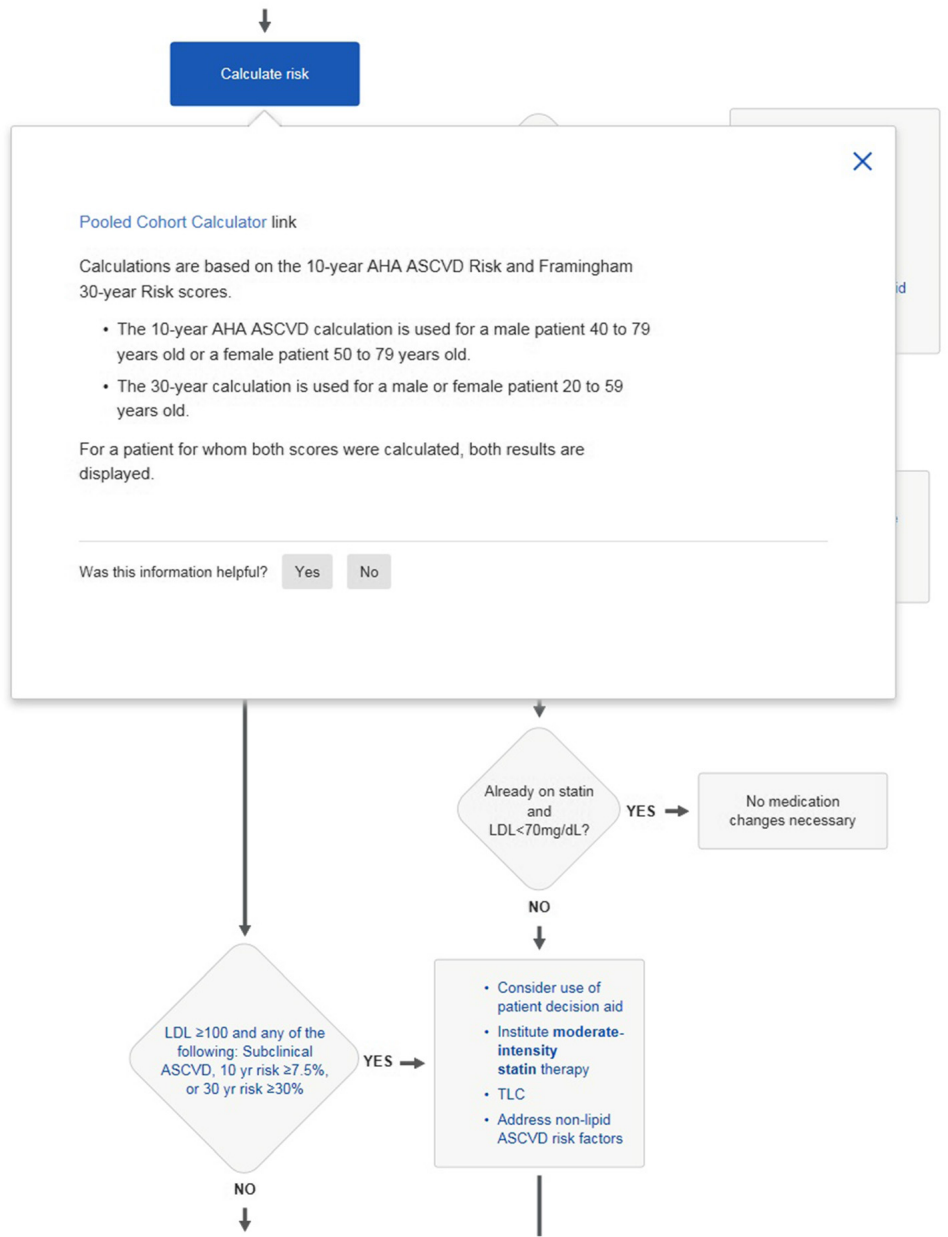
Section of lipid management care process model from Ask Mayo Expert. Risk calculation component of care process has been expanded

We chose to study care process models for atrial fibrillation, lipid management and heart failure because they incorporate risk assessment within the decision flow. For example the CHA_2_DS_2_VASc score in the atrial fibrillation care process model is used to decide about anticoagulation (Fig. 1); the American College of Cardiology atherosclerotic coronary vascular (AHA-ASCVD) risk score [5] is used for lipid decision making (Figs. 3 and 4). For heart failure the Seattle Heart Failure Model (SHFM) [9] is incorporated into the care process model. By quantifying the times taken with these care processes we could determine the time taken to complete the entire care process as well as determine the time for the risk assessment alone.

### Subject selection

We used a convenience sample of primary care health providers. From a total of 30 healthcare providers there were 23 primary care physicians, 3 nurse practitioners and 4 physicians in internal medicine residency training. All subjects had experience with the Mayo Clinic EHR and with use of care process models in Ask Mayo Expert.

### Scenario and task

We constructed a use case scenario that had three separate tasks. The scenario consisted of a patient with diabetes, congestive heart failure, and atrial fibrillation. The patient was a standardized test patient given to the providers on paper in text form. The providers were provided with the patient history, vital signs, lab values and pertinent imaging studies (Appendix). The scenario was presented as a paper sheet to the participants. The participants were asked to read the text and make a decision about anticoagulation, lipid management, and to make a decision about the benefit of an ICD. The click count and timing started when the participant started on the tasks. Since the case scenario was all in printed form, there were no clicks counted while the participant read the paper-based scenario. With the patient scenario information, the providers used the Ask Mayo Expert care processes for heart failure, atrial fibrillation and hyperlipidemia to calculate the risk scores and to identify potentially important patient treatment recommendations.

We asked the providers to do the following three tasks using the Ask Mayo Expert care process models:

1. Recommend treatment optimization for congestive heart failure (CHF) and show benefit with ICD using SHFM
2. Calculate the CHA_2_DS_2_VASc score and recommend treatment for atrial fibrillation
3. Calculate the AHA-ASCVD risk score and recommend treatment for hypercholesterolemia

To assess accuracy of the healthcare providers producing a numerical CHA_2_DS_2_VASc score and AHA-ASCVD risk, we compared the subjects’ scores with a reference standard obtained *a priori*. The test patient scenario was constructed to obtain a reference standard CHA_2_DS_2_VASc score of 4 and a reference AHA-ASCVD 10 year risk of 35 %. This calculated reference standard was verified by the first and senior authors (FN and RC) who used published calculators to confirm. The test patient scenario was also constructed to have a reference standard showing a survival benefit from ICD. This standard (survival benefit from ICD) was also confirmed from the test case data by FN and RC using published literature. Providers were labeled accurate in the tasks if they correctly calculated the CHA_2_DS_2_VASc score of 4, the correct AHA-ASCVD risk of 35 %, and indicated an ICD benefit.

### Clinician time estimate

We examined a sample of 49 primary care internal medicine provider panels totaling 30,792 patients. Using information from the primary care registry we were able to obtain counts of patients of the 30,792 who had vascular disease, diabetes, cholesterol of greater than 200, or last blood pressure above 135/85. We counted any patient having vascular disease, diabetes, cholesterol > 200, or blood pressure > 135/85 as a possible candidate for lipid management. Using this count of possible lipid management candidates we multiplied by the time it took for individual lipid management decision so that we could get an estimate of the time of lipid decision making for an entire primary care panel. With the same methodology we could also estimate the time lipid decision making would be per 1000 panel members.

We also used registry data to find the percent of paneled patients with congestive heart failure and atrial fibrillation. Using these percents, we could make similar estimates for the times of CHF and atrial fibrillation decision making per 1000 panel members.

### Data capture and analysis

We used Morae® Recorder software to collect the time it took to complete tasks and the number of clicks. The timing and click count started when the provider completed reading the paper text of the test patient scenario and stopped when the provider had completed the tasks (made a decision). The software allowed the times to be separated into the components we reported.

We used JMP 11.0 (SAS Institute, Cary, NC) for statistical analysis. The Shapiro-Wilk W test was used to compare the frequency distribution of times and clicks to a normal distribution.

## Results

Table 1 shows the mean time it took for providers to complete the tasks. This was separated into the risk calculation time and the combined risk calculation and clinical decision making time using the care process model in Ask Mayo Expert.

**Table 1:** Times and clicks for care processes and risk scores used for atrial fibrillation, lipid management, and heart failure (using Ask Mayo Expert)

Decision making task	Care Process Algorithm/Risk Score using Ask Mayo Expert®	Mean seconds to complete (SD), *n* = 30	95 % CI (Seconds)	*p* value, H0: secs to completion distribution = normal distribution (Shapiro-Wilk W Test)	Mean clicks (SD), *n* = 30	95 % CI clicks	p value, H0: clicks distribution = normal distribution (Shapiro-Wilk W Test)	Accuracy (%) of risk calculation or management decision, *n* = 30
Atrial Fibrillation	Management using CHA_2_DS_2_VASc	85 (18)	78 to 92	0.31	55 (24)	46 to 64	0.16	100
	CHA_2_DS_2_VASc calculation only	36 (9)	33 to 40	0.99	24 (6)	21 to 26	0.44	86
Lipids	Management using AHA-ASCVD	110 (32)	98 to 122	0.01	70 (25)	61 to 80	0.27	100
	AHA-ASCVD calculation only	45 (12)	40 to 49	0.006	18 (2)	17 to 19	0.003	90
Heart Failure	Management using SHFM Risk	347 (89)	314 to 380	0.04	159 (60)	136 to 181	0.38	100
	SHFM Risk calculation	171 (42)	155 to 186	0.70	63 (23)	54 to 72	0.45	N/A

For the 30 healthcare providers there were a total of 60 calculated scores (2 risk scores per subject: CHA_2_DS_2_VASc and AHA-ASCVD 10 year risk). From the 60 count sample there were 52 correct risk scores for 87 % correct. In the CHF decision, all 30 providers concluded that the test patient could benefit from an ICD (100 % correct).

There were 4 incorrect CHA_2_DS_2_VASc scores out of the 30, (13 %, 4 with score of 3 instead of 4) however since the threshold was a CHA_2_DS_2_VASc score of 2, none of these changes resulted in a change of decision making on whether or not to use anticoagulation. There were 3 incorrect calculations of the AHA-ASCVD 10 year risk calculation (10 %) but the incorrect calculations did not result in an incorrect statin recommendation.

**Fig. 5:**
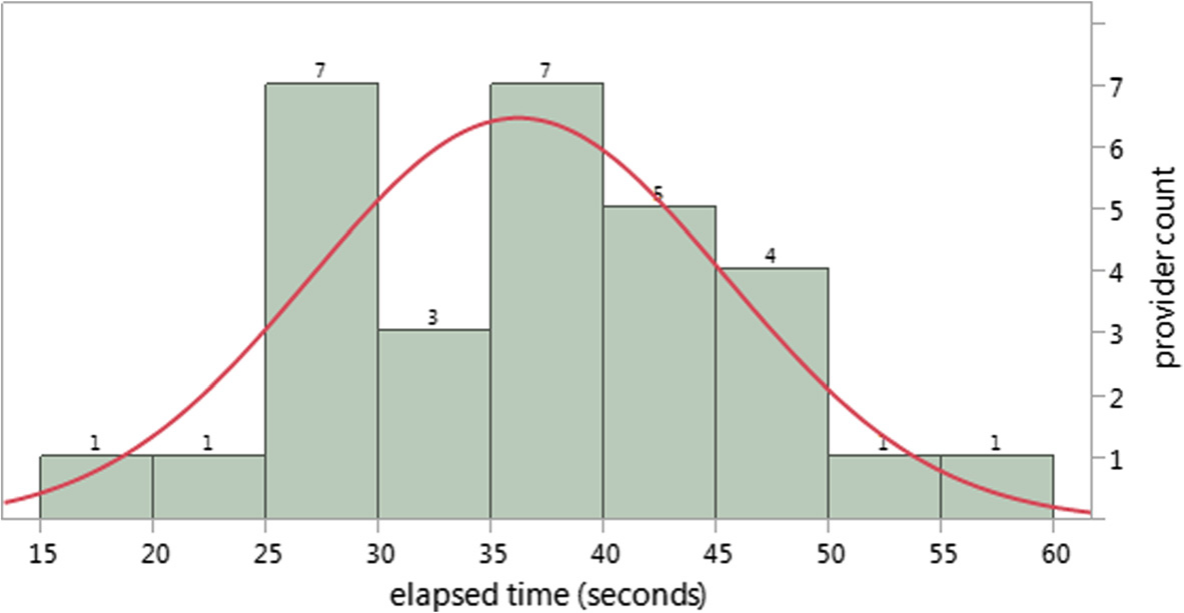
Frequency distribution of seconds elapsed to complete the CHA_2_DS_2_VASc. Histogram showing counts of providers by time to complete the CHA_2_DS_2_VASc with normal distribution curve superimposed. Goodness of fit with normal distribution: *p* = .99 (Shapiro-Wilk W Test)

**Fig. 6:**
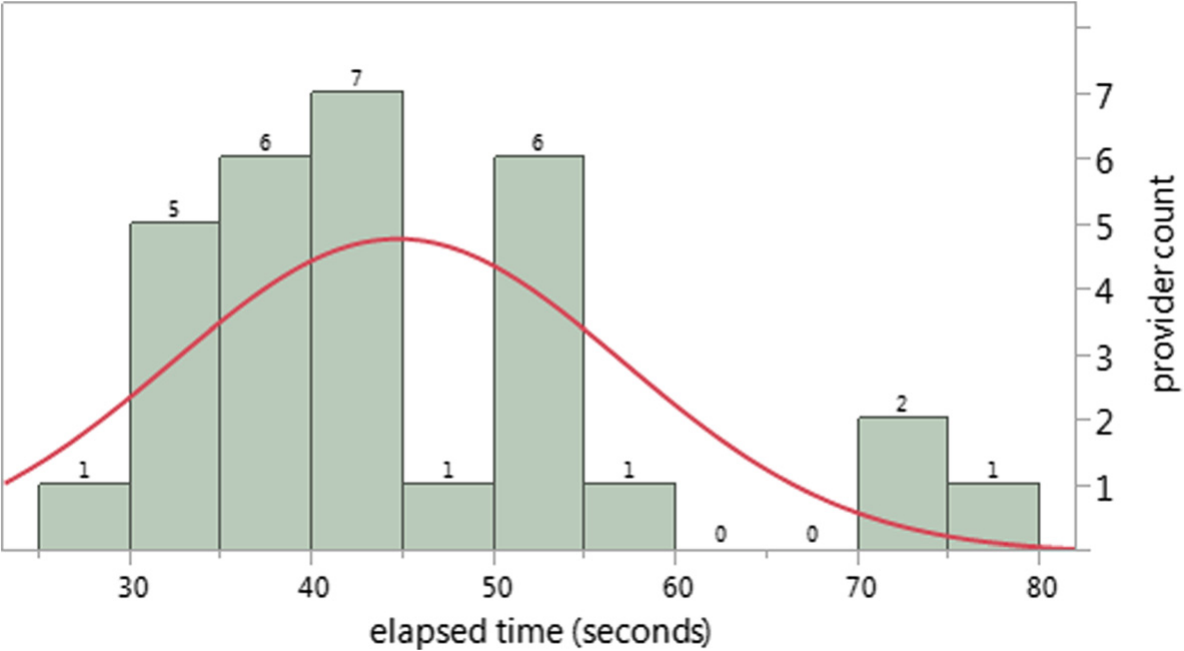
Frequency distribution of seconds elapsed to complete the AHA-ASCVD risk calculation. Histogram showing count of providers by time to complete the AHA-ASCVD risk with normal distribution curve superimposed. Goodness of fit with normal distribution: *p* = 0.006 (Shapiro-Wilk W Test)

Table 1 also has comparisons of the histogram frequencies of times and clicks to a normal distribution using the Shapiro-Wilk W Test. The histograms were consistent with normal distributions (Fig. 5) with the exception of the AHA-ASCVD risk scores (Fig. 6). Although there was individual variation in the times, there was only one statistical difference between groups when separated into a group of 23 staff physicians and a group of 7 others (3 nurse practitioners, 4 resident physicians). For the SHFM tool there was a statistically significant −44 s difference (CI 95 %; −6 s to −81 s) between groups; mean staff physician completion time was quicker at 160 s compared to the group of residents and nurse practitioners at 204 s. Although the 23 member staff physician group maintained an average advantage of 26 s over the 7 member nurse practitioner and resident physician group for the total CHF decision making, this difference was not statistically significant.

### Estimate of time involved in panel risk calculation

There were 30,792 patients in the 49 provider (42 physicians, 7 NP/PA) panels. The mean age of the patients was 57 with a median age of 58 and interquartile range from 42 to 71. Females accounted for 58 % (17,829) of the patients.

Tobacco users accounted for 8.8 % (2,707) and those with total cholesterol over 200 accounted for 27 % (8,447). Blood pressure was most recently recorded as ≥ 140/90 in 18 % (5,510) and glucose was last recorded ≥ 100 in 32 % (9,786). By combining these categories, there were 56 % (17,125) who either were tobacco users or had blood pressure ≥ 140/90, had cholesterol ≥ 200, or glucose ≥ 100.

Since elevated glucose, hypertension, hypercholesterolemia, and tobacco use all are associated with cardiovascular risk, we used the 56 % (17,125 patients) in the following calculation of total time it would take to examine the AHA-ASCVD risk and then decide on lipid management for this group. Using the average calculation time of 45 s for AHA-ASCVD risk calculation and 110 s for total lipid decision making, this would be 214 h and 523 h for calculating the entire panel cardiovascular risk and lipid management decision making, respectively. This also converts to a staggering 1.2 million mouse clicks to examine cardiovascular risk in lipid decision making for the 17,125 patients at higher risk.

In terms of individual providers, a provider with a panel of 1000 in our practice could expect about 7 h of time spent in calculating AHA-ASCVD risk scores and 17 h examining lipid guidelines based on the AHA-ASCVD risk score. He would have 39,000 clicks in this process.

Using similar calculations based on a 7.6 % prevalence of atrial fibrillation and 5.3 % prevalence of CHF in our primary care internal medicine practice, a provider with a panel of 1,000 patients could expect to spend 1.8 h for anticoagulation decision making on all his atrial fibrillation patients; 45 min of that would be used calculating CHA_2_DS_2_VASc scores. Our provider with 1,000 patients might also expect to spend about 5.1 h in decision making about the benefit of ICDs for all his CHF patients.

## Discussion

Clinicians may be spending a considerable amount of time on risk calculators for decision making. This study quantifies the time and clicks that cardiovascular risk assessment and decision making may take. Although the accuracy was 87 %, the inaccuracies did not cause a significant change in recommendations.

The time and effort that it takes to do risk assessments may be one of the major factors explaining why only 20 % of primary care providers routinely calculate risks [3]. Our estimated calculation of two minutes for a lipid management decision is a potential reason why these assessments are only done 20 % of the time.

Given the right rules engines and data sources, this type of clinical decision support could be placed into software. Evidence already shows that having this information readily available will result in improved guideline adherence and will improve care [3].

How do these times compare with other tasks that primary care providers perform? Our own internal time analysis of secure messages shows that the same providers generally can review patient laboratories and tests within the EHR and send a secure message in about 3 to 5 min. Another healthcare institution estimated time for preventive services during a visit for mammography, nutrition counseling, and exercise counseling at 2.54, 1.34, and 2.89 min respectively [13]. If time was saved by calculating risk and determining recommended care prior to the visit, there would be more time for shared decision making concerning that care. For example, after the decision has been made to initiate a statin, it takes time to convince the patient to actually purchase and take the drug. One study that examined consultation time for shared decision making for antithrombotic agents and atrial fibrillation showed a median of 31 min taken in shared decision making [14]. Likewise, it is no small task to explain to the patient what an ICD is, and why it is necessary. Reduction in time to make the initial decision will likely free up more time for substantive shared decision making with the patient. We already know that shared decision making has a positive impact for use and adherence to statins [15, 16]. Based on our study results, more automated calculation of risk scores and additional computerized clinical decision making should free up time at the point of care that could be used more productively.

Risk calculations are often an ongoing process as part of a periodic review. In the particular case of lipid management, a 10 year risk calculation is suggested every 4 to 6 years in patients without known cardiovascular disease [17]. For those with heart failure, an “annual heart failure review” is suggested to update prognosis [18]. The hours of time in our estimate for a practice panel are ones that would have to be repeated periodically. Practices using these results would need to consider these times to be a repeated expense rather than just a one-time proposition.

An advantage of our study is that we used software to capture the time intervals. We also captured total number of clicks with this technology. Using the different dimensions of burden including time and clicks we get a more complete view of clinician burden than has been previously published.

There are some limitations to the study. Ask Mayo Expert care process models were designed specifically for point-of-care decision making. The care process models allow providers to quickly find the care process on the intranet and move rapidly through the decision points of the process to find the recommended management. Also the care process models either have imbedded risk calculators or links to risk calculators so that providers do not need to search for them. Institutions without Ask Mayo Expert would likely take a different amount of time to complete the same tasks. In addition, our timings for atrial fibrillation decision making did not take into account a more complete risk and benefit analysis of anticoagulation. Although the HAS-BLED [19] scoring system is included in the Ask Mayo Expert care process model, we did not ask the subjects to obtain a HAS-BLED score so the time of a complete decision making process may be underestimated. Future studies on decision making will need to evaluate more complete care processes that may involve several risk scores involving a balance between treatment benefit and risk. The complexity of the task also does change the utility of decision support tools. Our study focused strictly on primary care providers. It is possible that these tools are not that helpful for certain providers who may have committed the CHA_2_DS_2_VASc scoring criteria to memory.

A limitation of our study design was we used a convenience sample; we did not have the resources for a larger group of participants that could be allocated to a control and intervention group. However, the lack of a control does not make this study irrelevant for the practicing clinician who already has some notion of the time it takes to do these tasks. In addition, informatics is rapidly enhancing clinical decision making. We are already in the process of automatically calculating CHA_2_ DS_2_Vasc scores and AHA-ASCVD 10 year risk scores from data already in the medical record. Our current results can serve as a potential benchmark to compare with further enhancements in decision making that involve lipid management, heart failure, and atrial fibrillation.

Another limitation is that this study examines the best case scenario in which the clinician has all the data required for the risk assessment at hand. Our scenario provided all the basic data needed for the risk assessments and clinical decision making. In a clinical setting there would not be a summarized sheet of paper with all the pertinent data to make the risk assessments and decision choices in the algorithms. A more realistic case would have the clinician searching through the medical record to obtain the baseline data such as lipid information, blood pressure, ejection fraction, QRS length, etc. Our timing data has to be interpreted in that regard. Real point-of-care times would not likely be as rapid because of the extra time involved in collecting the data needed for the risk assessment tools and the care process algorithms. Our participants were also a convenience sample. There was no *a priori* selection of participants by familiarity of use with this tool. With familiarity of the tool and multiple repetitions we expect that the times would diminish. Also, since the click counts started after reading the patient scenario, our click counts likely underestimate real point of care decision making where pertinent data is spread throughout the EHR.

These decision-making tools are less likely to be used in practices with younger and healthier patients. Internal medicine patient panels can be widely different from panels in family medicine. In our Mayo Clinic primary care practice in Rochester, Minnesota, the primary care internal medicine panel has a 7.6 % prevalence of atrial fibrillation while atrial fibrillation is only present in 1.4 % in the family medicine patient panel (*p* < 0.0001). For congestive heart failure, the prevalence is 5.3 % in our primary care internal medicine panel and 0.93 % in the family medicine panel (*p* < 0.0001). This difference in prevalence in atrial fibrillation and congestive heart failure would make the impact and importance of these particular decision-making tools potentially less in the family medicine practice. For example, based on the differences in prevalence of CHF in the internal medicine practice and family medicine practice, an internist with a panel of 1000 could expect to spend 5.1 h using the decision making tool for all his CHF patients, while the family medicine doctor with 1000 patients would only spend a little under an hour.

This study can be used by informaticists to examine how automatic risk calculators, pulling data from multiple sources, and use of clinical decision support could help speed the decision making process for the clinician. Those interested in provider workload can use this information to estimate clinical burdens on providers; those looking at population health can use these results to extrapolate clinician time to other populations.

At Mayo Clinic, we now have the AHA-ASCVD and CHA_2_DS_2_VASc risk scores automatically calculated for our primary care practice patients. We are in the process of seeing how this can help point-of-care lipid management and we anticipate a significant uptake of statin use as has been seen elsewhere when risk information was calculated prior to the visit [3].

Automatically calculated risk scores could be successfully used in population management. High risk patients could be identified and sent portal messages containing information about possible therapeutic options. For example, patients with high cardiovascular risk might be sent information about how their risk might be decreased with use of a statin. Different statin options could be explained prior to the visit. Patients with atrial fibrillation and an appropriate CHA_2_DS_2_VASc score could be sent information about different anticoagulation options, including newer anticoagulation medications and information about home anticoagulation monitoring.

## Conclusions

A significant amount of time is spent with risk calculation even under best circumstances. Our study demonstrates that under best case circumstances a provider in our practice would be spending an average of seven hours per 1,000 paneled patients just to calculate lipid-related risk. Generating individualized lipid risk-reduction recommendations would take an additional 10 h per 1,000 patients even with an accessible online care process algorithm (Ask Mayo Expert) and additional pertinent patient information readily available. Given the increasing time burden placed on physicians, we need to pursue increasing levels of computerized clinical decision support, including risk factor calculation and additional clinical support based on those risk calculations.

## Abbreviations

AHA-ASCVD risk, American College of Cardiology/American Heart Association - Atherosclerotic Cardiovascular risk; CHF, congestive heart failure; EHR, electronic health record; ICD, implantable cardioverter-defibrillator; NP/PA, Nurse practitioner/Physician assistants; SHFM, Seattle heart failure model

## Appendix

Case scenario used in this study for timing all participating physicians, nurse practitioners and physician assistants in risk assessment and clinical decision-making tasks (contains the information that each participant was to use for calculation of risk and in the care process models).

### Case scenario for risk assessment and clinical decision-making tasks

50-year-old African American female with a history of type II diabetes mellitus for 10 years. She also had hypertension for 5 years. She smokes 1 pack per day of cigarettes for the last 20 years, and consumed 1 glass of wine daily. She comes in today with a complaint of shortness of breath with mild exertion and has noted pedal edema for last four weeks.

Current medications: Metformin 1000 mg po bid; Glucotrol 5 mg po q daily; Atenolol 50 mg po daily; Ecotrin 325 mg daily; Zocor 10 mg po q hs daily.

Past medical history: No history of chest pain, myocardial infarction, peripheral arterial disease, transient ischemic attacks or stroke.

Examination: Weight is 120 kg; height, 5 ft 4 inches; blood pressure 150/94; pulse is 92, irregular. Jugular venous distention is increased by 6 cm. Heart examination shows S1, S2 is normal but irregular, S3 is positive. Lungs have crackles both bases. Bilateral 1+ pedal edema.

Labs: Fasting blood sugar 150; Hemoglobin A1c 7.5; lipid profile shows triglyceride 160; LDL cholesterol 98; total cholesterol 170; HDL cholesterol 40; non HDL cholesterol 122; creatinine 1.0; Na 140; K 4.0; hemoglobin 13.5; lymphocyte percent 40, SGOT 20. Na 140, uric acid level 6, BNP 600; albumin 4; TSH 4; Ca 9; Mg 2.2.

EKG shows Heart rate of 90, atrial fibrillation, QRS 110 mSec, nonspecific ST-T wave changes.

2D Echo shows left ventricle hypertrophy with generalized hypokinesia, ejection fraction 30 %. No regional wall motion abnormality or valve abnormality.

Chest x-ray shows increased heart size with pulmonary venous congestion.

Tasks

1. Review CHF (congestive heart failure) care process model from Ask Mayo Expert and recommend the appropriate treatment. The patient questions benefits of your recommendations re: CHF treatment. Review Seattle heart failure model and show benefit from treatment recommended for her. At this time, she asks, “What is an implantable cardioverter-defibrillator?” Will she need that too? When should she come back for follow-up, labs, and medication adjustment?
2. Review atrial fibrillation care process model from Ask Mayo Expert. Calculate the CHA_2_DS_2_VASc score. Recommend management for her atrial fibrillation.
3. Review lipid care process model from Ask Mayo Expert. Calculate AHA-ASCVD risk score and recommend treatment.
